# Relations and dependencies between morphological characters

**DOI:** 10.1007/s12064-017-0248-z

**Published:** 2017-05-31

**Authors:** Jürgen Jost

**Affiliations:** 1 Max Planck Institute for Mathematics in the Sciences, Leipzig, Germany; 2 Santa Fe Institute for the Sciences of Complexity, Santa Fe, New Mexico USA

**Keywords:** Morphology, Taxonomic character, Dependencies between characters, Structural constraints, Functional correlations, Tensor product, Fiber bundle

## Abstract

In biological classification, a character is a property of a taxon that can distinguish it from other taxa. Characters are not independent, and the relations between characters can arise from structural constraints, developmental pathways or functional constraints. That has lead to famous controversies in the history of biology. In addition, a character as a tool of data analysis has some subjective aspects. In this contribution, I develop algebraic and geometric schemes to address these issues in a mathematical framework.

## Introduction

Olaf Breidbach repeatedly asked the question how to define a character within the framework of morphology.^1^ Morphology is a concept introduced by Johann Wolfgang von Goethe^2^ as the science of structural relations. Goethe’s concept was not a static one, but depended on dynamic relations, metamorphoses. One of his aims was to derive all parts of a plant from a primordial or archetypical structure, the leaf. Within the context of a morphology in the sense of Goethe, a character, then is the appearance or outer realization of some inner property of an organism. As such, it is not an isolated feature, but rather reflects the structure of the organism as a coherent entity.

While in Germany, Goethe and others developed a natural philosophy underlying biological forms and Karl Ernst von Baer started the systematic investigation of animal development, embryology, in France; Étienne Geoffroy Saint Hilaire and Georges Cuvier developed competing concepts. The controversy between them erupted in the famous academy debate in 1830 (see Appel 1987) which the old Goethe followed with much interest. Georges Cuvier founded the subject of comparative anatomy, and he assumed that every animal was created according to its functional needs. He spoke of the conditions of existence, the conditions that were required for an animal to survive and reproduce in its environment. This lead to the principle of the correlation of parts which enabled him to recreate an entire animal (a *Chalicotherium*, from a mammalian line that died out in the pleistocene) from a single fossil bone. For him, there was no evolution, and new animals were created after catastrophic extinction events. There were four classes, embranchements, the vertebrate, articulate, mollusk, and radiate ones, between which no transitions were possible. His functional approach was thus based on teleological principles. For him, the main issue of zoology was classification, taxonomy, according to functional purpose, and for such a taxonomy, one should not just look at isolated characters, but rather identify the correlations between them according to functional, and thus for him, ultimately teleological principles.

Geoffroy, in contrast, considered every animal as a variation of a basic type, a bauplan, not unlike Goethe’s approach to plants. For that purpose, he developed his concept of analogy and later renamed as homology by Richard Owen in a somewhat different theoretical framework. In fact, in his approach which he called philosophical anatomy, Geoffroy even tried to link the four types, embranchements of Cuvier. In particular, he speculated about a systematic relationship between the vertebrate and articulate types. According to his theory, articulates were living inside their (exo) skeleton, instead of around an (endo) skeleton as the vertebrates, with a reversal of dorsal and ventral arrangements. Expressing his views in a caricature, a beetle was a turtle walking on its back.^3^ His approach was averse to taxonomy, but open to the possibility of evolution of a concrete animal form from a basic type. Since according to his principle of analogy, all the characters of a basic type could be found in all its representatives, albeit possibly in a modified or even hidden form and, perhaps, only detectable in early stages of embryonic development, one can only analyze different manifestations of the same character, but not compare animals according to different characters.

Both approaches, that of Cuvier as well as that of Geoffroy, did have some strong points, but also obvious difficulties. Goethe, when commenting about the debate (see Breidbach 2006 for a penetrating analysis), was surprised to see that two principles that he tried to combine, comparative osteology on one hand and a metamorphosis of nature on the other hand, were divided among opposing camps, but ultimately, his sympathy was with Geoffroy rather than Cuvier, and this was, in turn, triumphantly used by Geoffroy and his followers in the debate with Cuvier Appel (1987). Goethe tried to place his own work in comparative anatomy (the vertebral theory of the skull (also developed by the natural philosopher Lorenz Oken,^4^ with unclear priority, see, for instance, the references in Amundson (2005, p. 56) and the discovery of the intermaxillary bone in humans) into his framework of archetypes that was akin to that of Geoffroy.

For our subsequent analysis of characters and their correlations, Geoffroy’s approach is not directly useful, as it denies the emergence of new characters as distinctive characteristics of biological taxa, and in any case is averse to taxonomy as such. While Cuvier was thinking in terms of sharply delimited and discrete taxa, Geoffroy seems to have rather thought of a continuum of biological forms emanating from a general structural type. Nevertheless, a cladistic phylogenetic analysis Hennig (1966), which in many regards is closer to Geoffroy’s than to Cuvier’s way of thinking, is based on the distinction between shared and newly developed characters. Phylogenetic branchings according to the cladistic scheme are binary and reflected by the emergence of a single new character. Therefore, the correlation structure of characters in a cladistic scheme is very different from that in a functional scheme. We will return to that issue below.

After its initiation by Goethe, the speculations in the context of German Naturphilosophie and the controversy between Cuvier and Geoffroy, morphology more generally became the science of biological form. An excellent account, on which I shall also draw below, is given in Amundson (2005). As concerned with biological form, it was not so much about static forms, but about the formation and transformation of organic forms (Richards 1987). It, therefore, covered three aspects:

1. The formation of an organism by the repetition or variation of a single form. That was Goethe’s original approach. In addition, Owen’s concept of serial homology fits here. That aspect of morphology thus is about forms within an organism.
2. Comparative anatomy can study forms either in terms of their functions within the context of an organism that is adapted to its environment and, therefore, emphasize the correlations betweens the features of each organism as expressing its way of life–the approach of Cuvier (“conditions of existence”)–or it can explain the forms of taxonomically related organisms as variations of a basic type, a bauplan–the approach of Geoffroy (“unity of organic composition”).
3. Organismal forms can be understood as the results of embryonic development. This was the approach of von Baer. Similar developmental patterns should correspond to taxonomic relatedness.

From whatever perspective forms are compared, one needs criteria for such a comparison. Naturally, one will turn to specific properties or features of a form and/or the states of such features to distinguish it from others, or use internal relationships between the different parts to identify what is characteristic of one form. This leads us to the notion of a character. According to modern biological terminology, a character is a feature of an individual organism whose state distinguishes it from other organisms. These organisms could be from the same species or from another species or higher taxon. In the first case, the state of the character is individual and may play a social role; in the other cases, it can have a taxonomic role. Individual states of characters reflect the variation within a species. They can be heritable within lineages or the result of environmental, that is, non-heritable phenotypic variation. In this contribution, we are only interested in taxonomic characters. Such characters are the tools of biological classification. Ernst Mayr Mayr (1982), p. 185, defines: “*Classification is the ordering of organisms into taxa on the basis of their similarity and relationship as determined by or inferred from their taxonomic characters*” (Emphasis in the original). As defined in Futuyma (1998), p. 93: “In systematic studies, a character is a feature that is thought to vary independently of other features and to be homologous among the taxa of interest [...] A character state is one of the alternative conditions of the character. Thus, 'present' and 'absent' are two states of the character 'thumb' among primates. Likewise, a particular position in a DNA sequence is a character, the possible states of which are A, T, C, and G”. (Emphasis in the original). While I find some aspects of this questionable, as will become clear later, let us start to elaborate upon this definition. I shall speak of a taxonomic character in the sense of that definition. More precisely, a taxonomic character is one which is inferred to have existed in the most recent common ancestor of a taxon, even though it might not be present in all species derived from that common ancestor. For instance, limbs are a tetrapod synapomorphy, but not all tetrapods have limbs, e.g., snakes, many skinks etc. Therefore, Mayr includes the state “absent”, and with that provision, its states vary and thereby allow to distinguish the different taxa in a given systematic task. For concreteness, we consider different species here, but the same type of analysis applies, of course, also to higher taxonomic units.

Taxonomic character and their states should thus be heritable, or at least be acquired or produced by a heritable mechanism. The states of taxonomic characters should ideally be shared by all members of a species. This is not always the case, however. For instance, characters could be different between juvenile or larval and adult individuals, or between male and female ones. From a logical perspective, this can be easily handled–the character may consist in the larvae having such and such properties, or a species may be characterized by a particular form of sexual dimorphism. In paleontology, however, where the species are reconstructed on the basis of morphological characters and their states, such phenomena lead to well-known difficulties, and phenomena like sexual dimorphism can cause misclassifications. Mimicry can also fool species identification. Sometimes, characters are only expressed under certain circumstances. For instance, in the primate species *Pongo pygmaeus* (orang-utan), males that have acquired a dominant position that enables them to mate develop flanges (cheek-pads) that make them look different from other males (see MacKinnon (1974). The states of other characters, like plumage color or fur density, can vary between seasons. In addition, there may be pathological cases where characters are differently expressed. We should be careful here, however, and not lump phenomena together that biological theory wants to disentangle. Anticipating some of the subsequent discussion, character changes can be developmental or evolutionary. As we know since Karl Ernst von Baer, developmental patterns are regular, a particular developmental pattern can then also be used as a character. Not all characters need to be morphological. (For a recent analysis of the relation between animal development and morphology, see Minelli (2003). Evolutionary changes have a different conceptual role. For selection to work on some character, there needs to be some variation within a species (see, for instance, Breidbach and Jost 2004) for a species concept that incorporates such variation]. However, when the state of a character varies between the members of a species, it may not be suited for the identification of the members of that species.^5^ In addition, in evolutionary time, one can only track the change of a character within a species if that species can be consistently identified through other characters that stay constant.

Returning to the above quote from Futuyma (1998), but expressing it in slightly more abstract erms, when one wants to distinguish subclasses within a given class, the character should be shared by all subclasses of that class, but its value or state should vary between those subclasses. For the example of the position in the DNA, this depends on an alignment of the different DNA sequences, as otherwise a position cannot be consistently identified. This alignment, in turn, requires that other positions be invariant, that is, do not vary, in contrast to the position in question. Thus, here, a character cannot be consistently identified without a framework of other characters. In addition, the different positions in the DNA need not vary independently. We may, therefore, need to look at several positions simultaneously.

In any case, the concepts employed will and probably also should depend on the theoretical framework employed. In addition, since the middle of the last century, there are three different theoretical approaches to biological systematics (see, for instance, the surveys and discussion in Mayr 1981, 1982; Futuyma 1998). The phenetic approach introduced by Michener and Sokal (1957) argued for a classification on the basis of overall similarity, using as many features as possible, instead of only those that an individual taxonomist might feel most important in the classification task at hand. Of course, this naturally leads to the question if, and if so, how different features should be weighted. Therefore, it seems difficult to eliminate all subjective judgements and turn phenetic classification into a purely objective scheme. In contrast to the numerical or statistical perspective advocated by the phenetic approach, the cladistic method of Hennig (1966) is a scheme of binary logical division. The key argument is that synapomorphies, that is, shared derived character states, characterize monophyletic taxa, and only such taxa, the clades, are admitted in his cladograms. When different characters suggest conflicting taxonomies, the principle of parsimony is applied. The evolutionary systematics, advocated in particular by Mayr (1982), admits the logical value of the cladistic scheme, but points out certain practical difficulties, resulting, for instance, from homoplasies or convergences. Furthermore, Mayr (1981) also advocates that not only branching, but also subsequent evolutionary divergence should be reflected in the classification. He, therefore, concludes that concrete biological contexts might require some modification of the cladistic scheme. An often cited example is the birds which according to the cladistic scheme should be grouped together with the crocodiles as a subgroup, consisting of extant archosaurs, of the reptiles. Else, the reptiles become a paraphyletic group. Since, however, the birds have undergone so profound evolutionary changes (in cladistic terminology, their autapomorphies outnumber the synapomorphies with the crocodiles), in an evolutionary classification, according to Mayr, they should be granted a higher taxonomic level. (Modern taxonomists, however, mostly no longer agree with Mayr on this point).

While the declared purpose of Hennig’s cladism is to link taxonomic classification with phylogenetic relations and the purpose of Mayr’s classification is to reflect the evolutionary history, for the phenetic approach, these bonds are not so tight. Therefore, from the phenetic perspective, the concepts are less burdened with theoretical weight and can thus be developed more freely. For that reason, in this contribution, the technical part will be mainly expressed in phenetic terms, although this should not be interpreted as taking sides in the dispute between the different approaches to taxonomy. In addition, in any case, a lot of biological theory will inevitably enter.

For the purposes of this contribution, a character is thus a combination of features of a biological taxon whose states can distinguish it from other taxa. Thus, it has a taxonomic function, the distinction between different taxa. This brings in a relative aspect. A character is not an absolute feature. For instance, carbon-based metabolism or DNA-based replication or, more specifically, the four nucleotides and the 20 different amino acids that are found in cells are not characters that are taxonomically useful, because all biological cells, and hence, all organisms possess them (we are not concerned with viruses here). In addition, subjective aspects enter, because a character is a feature combination that is selected, for instance, by a paleontologist that wants to classify fossils, on the basis of its recognizability, identifiability, stability, etc. In addition, most characters concern shapes, that is, ultimately visual patterns, and thinking in images is a powerful mental tool with its own inner structure. This aspect is explored in Breidbach et al. (2011).

Nevertheless, for biological theory, a character cannot be completely subjective. It should be based on form, function, or process of generation. The form could be its geometric shape, its physical properties, or its chemical composition. A function can only be assigned within the wider perspective of a living and reproducing organism. Generation can be understood in developmental or evolutionary terms. Thus, all the basic components of biological theory enter, and since there has been considerable tension between those components in many debates in biological theory, like those between Georges Cuvier and Étienne Geoffroy Saint Hilaire, or between Richard Owen and Charles Darwin, the notion of a character can easily collapse under a lot of theoretical weight.

Essentially, we need to address the tension between the fundamental biological fact that organisms are not aggregates of features, but functionally and developmentally integrated wholes, and the requirement of systematic analysis to decompose such an organismal whole into character data (see Kearney 2007). Of course, this is not a strict dichotomy, as decomposability of the phenotype is not only an aid for taxonomy, but also an important feature of the biology of the organisms. Quasi-independence of characters/features is necessary for adaptation of different functional systems, i.e., the fact that the sensory organs can evolve quasi-independently of the locomotory organs, etc. Hence, the proper decomposition of the organism in systematics shall follow the natural variational degrees of freedom that allow adaptive evolutionary change. It is, in fact, an aim of this article to approach such decomposibility from a mathematical perspective.

From a phylogenetic perspective, these character data should indicate phylogenetic relationships. They should be evolutionary homologues for grouping taxa together or reflect changes in particular lineages for distinguishing taxa. Of course, one needs to avoid circularity here and carefully address the relation between observed patterns and structures and the explanatory process that is supposed to have created them.

Wagner and Laubichler (2000) and Wagner and Stadler (2003) develop a notion of a character based on the genotype–phenotype map. In their theory, characters correspond to local factors in phenotype space. The latter space inherits its mathematical structure from the genotype–phenotype map and the definition of a local factor, therefore, requires some conceptual mathematical framework which they develop (Stadler et al. 2001; Wagner and Stadler 2003). In essence, a character is thus a part of the phenotype that can be changed by modifications of the genotype independently of other features of the phenotype. Even with the advances of modern omics biology and bioinformatics, however, both the principles and the details of the genotype–phenotype map are not yet completely understood, even though insight from many detailed examples is rapidly accumulating. And in many contexts of morphology-based research, as in paleontology, one typically does not have access to genetic data, but needs to work with phenotypes or with what is preserved of them in the fossil record. Therefore, while the approach of Wagner, Laubichler, and Stadler may well represent the ultimate solution of the problem of the character definition in terms of biological theory, here, we propose a rather different approach that is based on principles of data analysis. This approach will draw upon scientific constructions that are not biological in nature, but hopefully, our approach can still shed some new light on the issue. Of course, we shall also try to place it into the context of biological theory.

We shall emphasize in particular the correlation structure of characters. Wagner’s concept of character identity networks (Wagner 2014) seems to go in a similar direction, but as already mentioned from a different, and more biological, perspective.

We should also point out that in this contribution, we do not address the statistical aspects of phylogenetic inference, although in practice, these must be combined with the algebraic and geometric schemes that we shall present below. Originally, this had met with resistance from cladistic phylogenetic analysis (see Felsenstein 1994 for pointing out the importance of statistical criteria), but this is now generally accepted and widely applied (for modern methods, see, for instance, Caspi et al. 2005).

## Morphology

Let us first recall that part of the analysis of (Breidbach and Jost 2006) that is relevant for the present purposes. Morphology as a biological discipline is concerned with the structure and relatedness of different organisms (see the entry “Morphologie” in Toepfer 2011 for a good survey). For that purpose, as already explained in the introduction, morphologists need to identify features that characterize one class of organisms and distinguish it from others. Such features are called characters, because they are supposed to be characteristic of such a class. When it comes to defining and identifying characters, morphologists are confronted with the problem that even two individuals from the same species may exhibit rather drastic variations, like the different races of domestic dogs. With respect to certain features, a particular dog may rather look like a cat than a dog from another race. Nevertheless, morphology has developed and possesses systematic criteria allowing for a distinction between feline and canine carnivores. These criteria, however, do not consist in single isolated properties, but have to take the entire structures and the relations between their parts into account. Thus, one has to use relative criteria, like relations between sizes of different body parts, or their relative position within the anatomical structure. This offers the advantage that we can compare body parts of different shapes in different animals according to their relative positions, see, for instance, (Kutsch and Breidbach 1994). A famous example was that Goethe could use such a comparative analysis on the basis of relative positions to establish a relationship between the temporo-mandibular joint of some mammals and the arrangement of the ossicles in the human ear.^6^

The question then arises whether this relative characterization can be formalized. Such attempts, however, have met with opposition from paleontologists. About a 100 years ago, the paleontologist Adolf Naef contrasted the intuitive insight of a morphologically trained scientist with analytical approaches which he, therefore, rejected (Naef 1919). An analytical clarification of the relative structural properties was not achieved at that time. Today, the situation looks hardly any better. A modern textbook in paleontology like (Benton 2015) gives up on this question and simply states “There are no objective rules of what is and is not a character” (p. 37) and, therefore, makes no attempt at a definition.

Of course, there have been some systematic attempts at this question. An isolated figure in the history of biological thought is Thompson (1917) who wanted to capture the corresponding relational regularities in a mathematical framework. Thompson transformed the shape of one organism into that of another organism by a geometric deformation. He argued in terms of visual patterns and thus could not transcend the intuitive level and achieve an analytical framework underlying the pictures that he used for the illustration of his ideas. However, when the similarity relations that are central for comparisons cannot be expressed analytically, there is no foundation for morphology. In any case, no biologically meaningful ’character’ can be extracted from Thompson’s drawings.

Thompson’s approach still falls into the predarwinistic typological framework of an idealistic morphology (see, for instance, Toepfer 2011, Vol. 2, entry “Morphologie”, p. 633). However, approaches with a different theoretical foundation do not fare much better concerning the problem.

Seilacher’s morphodynamics (Seilacher and Gishlick 2015) constitutes a more refined approach while sharing some conceptual similarities with Thompson’s coordinate deformations. For him, morphogenesis rests on the basis of phylogenetic relations, but unfolds through the interaction of biological function, environmental pressure, and self-organizing pattern formation, with an emphasis on the latter, that is, mechanical or chemical forces that are not genuinely biological, but are thought to operate also in other domains.

## Algebraic representations of correlations

As Wagner and Stadler (2003) point out, we need to distinguish between independent generation from the genotype—which is the basis of their definition—and independent variation of characters. Typically, characters, even though the genetic mechanisms of their generation might be independent, are correlated as they depend on each other to secure an organism’s function. Actually, the issue is more intricate than that. There exist many genetic control and regulation mechanisms, at DNA, RNA, and intercellular level, that secure a coordinated expression of genes that produce phenotypic traits, features, structures, etc., that belong together for a functional organism. At a different level, there are also structural constraints that link the expression of different characters. For instance, the forehead could only grow, without leading to mechanical instabilities, and make a substantial increase of the prefrontal cortex possible in human evolution, because the size of the jaws was reduced, which, in turn, was triggered by nutritional changes (for a review of human cultural and biological origins, see Klein 2009). More fundamentally, the bauplan of a taxonomic class imposes constraints on the relations between characters. These constraints need not be absolute. There exist examples where some species in a taxonomic class deviate in a drastic manner. For instance, *Wolffia* are tiny flowering plants that are freely floating on water and do not possess roots, leaves, or a stamen.

To approach the issue of independence of characters more systematically, we now make a shift in perspective and terminology and look at features. A feature is something that can be measured or counted or checked for its presence. The aim behind this shift is that subsequently, we wish to identify characters as suitable combinations of features.

To formalize the issue of the dependencies between features, we shall now develop an appropriate mathematical framework (for mathematical background, see Jost 2015). This framework shall utilize products, but instead of Cartesian products as in (Wagner and Laubichler 2000; Wagner and Stadler 2003), we shall work with tensor products.

Cartesian products are appropriate when we want to identify independent sets of features for a given carrier, but tensor products are useful when we want to express dependencies between carriers and features. In fact, from an algebraic perspective, Cartesian products are more like sums, whereas tensor products are true products.

The Cartesian product of two sets *A*, *B* is simply the set of all ordered pairs,

1 $$\begin{aligned} A\times B =\{(a,b):a\in A, b\in B\}, \end{aligned}$$

and it usually inherits whichever structure that the sets *A*, *B* additionally carry. For instance, if they are topological spaces, so is their product. For the tensor product, in contrast, the factors should be vector spaces, or more generally, modules over a ring. We shall be interested in the following instances. When a feature can be measured, we assume that measurements are taken as real numbers, that is, with values in ${\mathbb R}$ (or some subset, therefore, like the positive reals). We then consider vector spaces over the field ${\mathbb R}$. When features are counted, the possible values are taken in the ring of integers ${\mathbb Z}$ (or again some subset, like the positive integers). Since we may consider ${\mathbb Z}$ as a subring of the field ${\mathbb R}$, fortunately, we do not really have to invoke details of commutative algebra beyond the elementary theory of vector spaces. Finally, when we simply check whether a feature is present or absent, we take the value 1 for presence, 0 for absence, and, therefore, work with the field ${\mathbb Z}_2$ consisting of these two values with addition and multiplication defined modulo 2. In any case, the algebraic structure of the field will not play an essential role in most of our constructions.

Thus, if *V* and *W* are vector spaces (finite dimensional, for simplicity) with bases $e_i, i=1,\ldots ,n,$ and $f_j,\,j=1,\ldots ,m$, respectively, then their tensor product has a basis consisting of objects $e_i \otimes f_j$, and

2 $$\begin{aligned} V\otimes W=\left\{ \sum _{i=1,\ldots ,n, j=1,\ldots ,m} c_{ij} e_i \otimes f_j:\, c_{ij} \in {\mathbb R}\right\}. \end{aligned}$$

Thus, when the dimension of *V* is *n* and that of *W* is *m*, then the dimension of $V\otimes W$ is *nm*, whereas the Cartesian product $V\times W$ has dimension $n+m$.

Of course, either product structure naturally extends to more than two factors.

To illustrate how one and the same biological structure is represented differently depending on which type of product that we employ, let us consider a situation where we have *n* different carriers, for instance taxonomic units, like species, and *m* different features that can be measured in terms of real numbers, like the lengths of certain appendices. When we count or check instead, we simply replace ${\mathbb R}$ by ${\mathbb Z}$ or ${\mathbb Z}_2$ in the subsequent formalism.

When $c_{ij}$ is the measurement of the *j*th feature for the *i*th carrier, then, for fixed $i_\star$, $c_{i_\star j}, j=1,\ldots ,m$ is the vector of feature values for carrier $i_\star$, and when $j_\star$ is fixed instead, then $c_{ij_\star }$ is the vector of the values of the fixed feature $j_\star$ across the different carriers. When we want to represent this structure in terms of Cartesian products, we should either form

3 $$\begin{aligned} \underbrace{{\mathbb R}^m \times \cdots \times {\mathbb R}^m}_{n\, \mathrm {times}}, \end{aligned}$$

when we want to look at the feature vectors for the different carriers, or

4 $$\begin{aligned} \underbrace{{\mathbb R}^n \times \cdots \times {\mathbb R}^n}_{m\, \mathrm {times}}, \end{aligned}$$

when we want to compare the feature values across the different carriers. Thus, in (3), a factor corresponds to one carrier, and in (4), a factor corresponds to a feature. In particular, in either case, we can single out one factor, in (3) to contemplate the feature values for a single carrier $i_\star$, or in (4) for the distribution of the values of a single feature $j_\star$ across the different carriers.

Before proceeding to the alternative representation via tensor products, let us make the following remark. While the carriers might be taken as given (perhaps, after some preliminary sorting of the data which are not of interest here), the *m* feature vectors can be rearranged by taking linear combinations (in Sect. 4, we shall also consider rearrangements that need no longer be linear). In particular, we could identify the most prominent directions for the variations of the feature values via principal or independent component analysis, which is one of the most basic techniques of data analysis (see, for instance, Bishop 2006). Here, however, we are less interested in the variability of features, but rather in dependencies between them.

For that purpose, instead of using Cartesian products, we represent the space of feature values for the different carriers as the tensor product ${\mathbb R}^n \otimes {\mathbb R}^m$. We can tabulate the values as a matrix

5 $$\begin{aligned}&\quad \,\,\,\,\, {\scriptstyle j=1} \qquad \dots \quad {\scriptstyle j=m}\nonumber \\ \begin{array}{c} {\scriptstyle i=1}\\ \\ \vdots  \\{\scriptstyle i=n} \end{array}&\quad\left( \begin{array}{cccc} c_{11}&{} c_{12}&{} \ldots &{}c_{1m}\\ c_{21} &{} &{} \dots &{} \\ &{} \vdots &{} &{} \\ c_{n1} &{} &{} \dots &{}c_{nm} \end{array} \right) \end{aligned}$$

where the *n* rows correspond to the different carriers and the *m* columns to the various features.

This representation allows us to easily express dependencies. The number of independent features is equal to the number of linearly independent rows, or, what is the same, the number of linearly independent columns. For later purposes, we express this in terms of tensor products. Let $a =\sum _{i=1, \ldots ,n}a_i e^i$ be a vector in ${\mathbb R}^n$, $b =\sum _{j=1, \ldots ,m}b_j f^j$ a vector in ${\mathbb R}^m$, where $e^1,\ldots ,e^n$ and $f^1,\ldots ,f^m$ are the standard basis vectors of ${\mathbb R}^n$ and ${\mathbb R}^m$, respectively. Thus, an upper index identifies a vector. Then, $a\otimes b$ is the matrix $C=(c_{ij})_{i=1,\ldots ,n, j=1,\ldots ,m}$ with entries $c_{ij} =a_i b_j$. Not every matrix can be represented this way. The general form is

6 $$\begin{aligned} C=\sum _{\nu =1,\ldots ,r}a^\nu \otimes b^\nu\, \text { with }\, a^\nu \in {\mathbb R}^n, b^\nu \in {\mathbb R}^m, \end{aligned}$$

for some $r\le \min (n,m)$. Here, again, the superscripts distinguish different vectors, while the subscripts have been used to indicate the components of a single vector. The minimal number *r* for which a matrix *C* admits a representation of the form (6) is called the rank of *C*.

7 $$\begin{aligned} \left( \begin{array}{ccc} 0&{}1&{}0\\ 1&{}0 &{}0\\ 0&{}0&{}1 \end{array} \right) = (0,1,0)\otimes (1,0,0)+(1,0,0)\otimes (0,1,0)+(0,0,1)\otimes (0,0,1) \end{aligned}$$

has rank 3. Here is an example of rank 1,

8 $$\begin{aligned} \left( \begin{array}{ccc} 1&{}2 &{}5\\ 2&{}4&{}10 \end{array} \right) = (1,2)\otimes (1,2,5). \end{aligned}$$

In this case, all the feature values are correlated, with a factor for each carrier. Thus, when we have a representation as in (6), with minimal *r*, we call each $b^\nu$ an independent feature combination, and the corresponding $a^\nu$ a vector of carrier factors. We can turn this into a preliminary

### **Definition 1**

A character cluster of a carrier-feature matrix *C* is an independent feature combination. Those features then are the characters representing the cluster.

Thus, what is identified as a character depends on the carriers under consideration. This makes sense, because the purpose of a character is to distinguish between different carriers. From each cluster, it suffices to consider one character, as the others are correlated with it and, therefore, do not contribute to further distinctions.

In general, an $m\times n$-matrix will have rank $\min\,(m,n)$, that is, the maximally possible one. In particular, when there are measurements errors, a feature matrix as above will typically have maximal rank. Nevertheless, we can try to approximate such a matrix by lower rank ones, within specified error bounds, to estimate the number of independent features within such error bounds. Therefore, we need to modify the above definition, prescribe some measurement error bound $\varepsilon > 0,$ and consider independent feature combinations of matrices $C'$ with $\Vert C-C'\Vert < \varepsilon$ that have minimal rank under this constraint.

We now turn to correlations between features and consider the situation of binary features. Such a feature may be either present or absent. Again, this is an idealization, and more complex cases can be handled within the framework developed here, but once more, we want to stay mathematically as simple as possible. We consider those features across many carriers, and when the feature *j* occurs with frequency $r_j$, we consider the relative frequency $p_j=\frac{r_j}{\sum _{\ell =1,\ldots ,m}r_\ell }$. Likewise, we have the relative frequency $p_{jk}$ for the joint occurrence of the features *j* and *k*. This yields the matrix

9 $$\begin{aligned} (p_{jk})_{j,\,k =1,\ldots ,m} \end{aligned}$$

which is symmetric ($p_{kj}=p_{jk}$) and whose row or columm sums give the individual relative frequencies ($\sum _{k=1,\ldots ,m}p_{jk}=p_j, \sum _{j=1,\ldots ,m}p_{jk}=p_k$). Again, we may try to represent this matrix as sums of tensor products. The special case

10 $$\begin{aligned} (p_{jk})_{j,\,k =1,\ldots ,m}=p\otimes p, \text { with }\;p=(p_1,\ldots ,p_m), \end{aligned}$$

now means that for each *j*, *k*

11 $$\begin{aligned} p_{jk}=p_j p_k, \end{aligned}$$

that is, the feature frequencies are uncorrelated. We can also form higher order tensors

12 $$\begin{aligned} (p_{j_1 \ldots j_s})_{\, j_1,\ldots j_s =1,\ldots ,m}\quad \qquad\text { for }\; 1\le s \le m, \end{aligned}$$

and if for each *s*

13 $$\begin{aligned} (p_{j_1 \ldots j_s})_{\, j_1,\ldots j_s =1,\ldots ,m} =p\otimes \cdots \otimes p \quad (s\text{ times}), \end{aligned}$$

then the feature frequencies are independent. Thus, in contrast to the above situation where we have analyzed the distributions of features among carriers where the minimal rank was realized for the case of complete dependence, here, when we consider the joint frequencies of features, minimal rank is achieved in the case of complete independence. More generally, dependencies between features lead to independencies between probabilities.

Again, we can interpret the ranks of the tensors $(p_{j_1 \ldots j_s})_{\, j_1,\ldots j_s =1,\ldots ,m}$ in terms of dependencies between the distributions of the features, see (Landsberg 2012). Now, however, a more subtle phenomenon can occur. It is possible that while the full tensor $(p_{j_1 \ldots j_m})_{\, j_1,\ldots j_m =1,\ldots ,m}$ has some rank *r*, it can be represented as the limit of tensors of rank $<r$. In mathematical terminology, this means that its border rank, the minimal rank of all the tensors of a sequence $P_N$ approximating *P*, could be smaller than the rank of *P* itself. This means that *P* contains dependencies that are not contained in the approximating tensors $P_N$. Typically, however, the rank of *P* equals its border rank, that is, the dependencies in *P* are robust against perturbations.

Given a matrix $C=(c_{ij})$ as above, we can form a frequency matrix *P* with coefficients

14 $$\begin{aligned} p_{ij}=\frac{1}{Z}\sum _k c_{ik}c_{j\,k}\, \text { with }\, Z=\sum _{r,s,t}c_{rt}c_{st}. \end{aligned}$$

### **Lemma 1**

*The rank of P is the same as that of C.*

### Proof

In fact, if *C* has rank *r* and

15 $$\begin{aligned} C=\sum _{\nu =1,\ldots ,r}a^\nu \otimes b^\nu \end{aligned}$$

as in (6), then

16 $$\begin{aligned} ZP=  {} \,C\cdot C^\star \quad \text { where }\; C^\star\; \text { is\, the\, transpose\, of }\,C\end{aligned}$$

17 $$\begin{aligned}= & {} \left( \sum _{\nu =1,\ldots ,r}a^\nu \otimes b^\nu \right) \left( \sum _{\mu =1,\ldots ,r}b^\mu \otimes a^\mu \right) \nonumber \\= & {} \sum _{\nu ,\mu }b^\nu \cdot b^\mu (a^\nu \otimes a^\mu )\nonumber \\= & {} \sum _{\nu ,\mu }a^\nu d_{\nu \mu }a^\mu \quad \text { with }\;d_{\nu \mu }=b^\nu \cdot b^\mu \nonumber \\= & {} \sum _{\nu =1,\ldots ,r} a^\nu \otimes e^\nu \quad \text { with }e^\nu =\sum _\mu d_{\nu \mu }a^\mu . \end{aligned}$$

Thus, the rank of *P* is not larger than *r*, the rank of *C*.

To get the converse, we reverse the steps leading to (17). We first observe that we may assume that the $a^\nu$ in (15) are linearly independent, as otherwise, the number *r* of summands would not be minimal. (If we had, for instance, $a^1=a^2=a$, then $a^1\otimes b^1 + a^2 \otimes b^2= a\otimes (b^1+ b^2)$, and we had reduced the number of summands). If we then have a representation (17) with a symmetric *P*, each $e^\nu$ has to be a linear combination $e^\nu =\sum _\mu d_{\nu \mu }a^\mu$ of the $a^\mu$, with symmetric coefficients $d_{\nu \mu }=d_{\mu \nu }$. We then find vectors $b^\nu$ with $d_{\nu \mu }=b^\nu \cdot b^\mu$. In fact, we can take the $b^\nu$ as the columns of a (symmetric) square root *B* of the matrix $D=(d_{\nu \mu })$, that is, $B\cdot B=D$. We then get $ZP= \left( \sum _{\nu =1,\ldots ,r}a^\nu \otimes b^\nu \right) \left( \sum _{\nu =1,\ldots ,r}a^\nu \otimes b^\nu \right) ^\star$, and $\sum _{\nu =1,\ldots ,r}a^\nu \otimes b^\nu ,$ then has to agree with *C* up to some global factor, a matrix *W* satisfying $WW^\star =\mathrm {Id}$, as *C* is determined by (16) up to such a factor. $\square$

Expressing the preceding in more verbal terms, dependencies between features make the relative frequencies independent, and conversely. Let us consider examples. Again, the rows will stand for carriers and the columns for features.

18 $$\begin{aligned} \left( \begin{array}{cccc} 1&{}0 &{}0&{}1\\ 1&{}0 &{}0&{}1 \end{array} \right) . \end{aligned}$$

Here, whenever a feature is present in the first carrier, it is also present in the second, and conversely. The relative frequency or probability $p_1$ of the first feature is 1, because it is present in all carriers, and so is the probability $p_4$ of the fourth one. Their joint probability $p_{14}$ is also 1, because they both occur in all carriers. Hence, $p_{14}=p_1 p_4$, and similarly for the other features ($p_2=p_3=0$, because they never occur). In this example, there are dependencies between the carriers and their feature combinations. In fact, the two rows of (18) are identical. If, in contrast, we had

19 $$\begin{aligned} \left( \begin{array}{cccc} 1&{}0 &{}0&{}1\\ 0&{}1 &{}1&{}0 \end{array} \right) , \end{aligned}$$

then there are no longer such dependencies between carriers and features, but we now have dependencies between the features themselves. Whenever we observe the first feature, we also find the fourth, and when the first is absent, so is the fourth. We have $p_1=p_4=.5$, because they are both present in half of the carriers, and for the same reason, also $p_{14}=.5$, and so $p_{14}\ne p_1 p_4=.25$ and similarly for other feature combinations. When we observe the first feature, the second is absent, and when the first is absent, the second is present. The carriers are independent, in the sense that their features are not correlated, but there now exist dependencies between the features.

The preceding construction might reveal some shortcoming of the preliminary Definition 1. The point is that there can also exist higher order dependencies between features, and the full tensor $(p_{j_1 \ldots j_m})_{\, j_1,\ldots j_m =1,\ldots ,m}$ can have a larger rank than the matrix $(p_{ij})_{i,j=1,\ldots ,m}$ of pairwise correlations. However, in binary branching, phylogenetic trees based on synapomorphic characters are as in cladistic analysis which cannot happen.

## Geometric representations of correlations

We shall now represent correlations in a geometric instead of an algebraic manner, using the mathematical language of (pre)sheaf theory. For the mathematical background and further references, see (Jost 2015). We again consider carriers $i_1,\ldots ,i_n$ and the features that they possess. For purposes of illustrations, we assume that each feature can only attain finitely many possible values, although the mathematical theory is not restricted in that manner. Not every carrier *i* need to possess all features *j*. For instance, for organisms without wings, the wing length is not a feature. We call the set of all occurring features the base space $B=\{j_1,\ldots ,j_m \}$. For each $j\in B$, we let $F_j$ be the set of possible feature values and call it the fiber over *j*.

For each carrier *i*, we then have a value $f_j(i)$ for those features *j* that *i* possesses. We call this assignment of feature values for carrier *i* the partial section $s_i$ defined by *i*. We add the qualifier “partial” here, because *i* need not possess all the features in *B* and, therefore, will have no values for those it does not possess. A (partial) section *s* assigns to each element *j* of the base *B* (when it is defined at that element) an element *s* (*j*) in the fiber $F_j$ over *j*. When a feature values is assigned to every feature in the base, we speak of a global section, or a section for short.

Thus

20 $$\begin{aligned} f_j(i)=s_i(j). \end{aligned}$$

In the left-hand side, where the feature *j* is the index and the carrier *i* the argument, we look at the various values of feature *j* that different carriers have. In the right hand side, where now the carrier is the index and the feature is the argument, we take a carrier *i* and look at the values for the different features that it has.

**Fig. 1:**
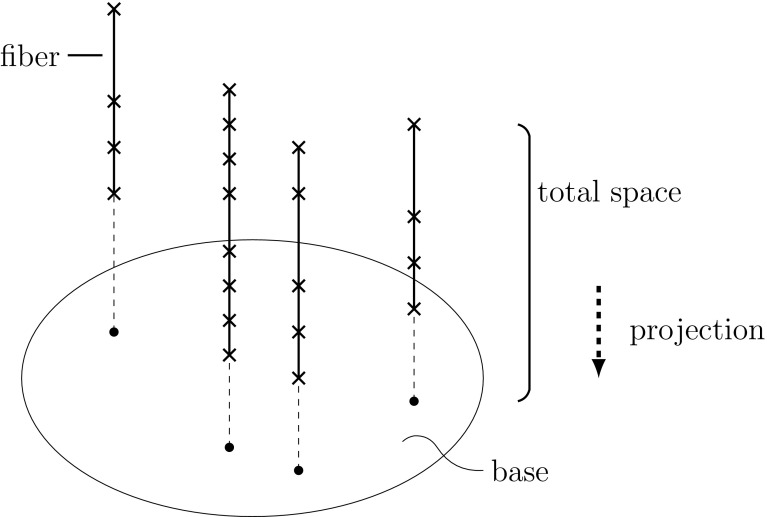
Base space consisting of the various features, the fibers with the possible feature, and the projection from the feature values to the features

**Fig. 2:**
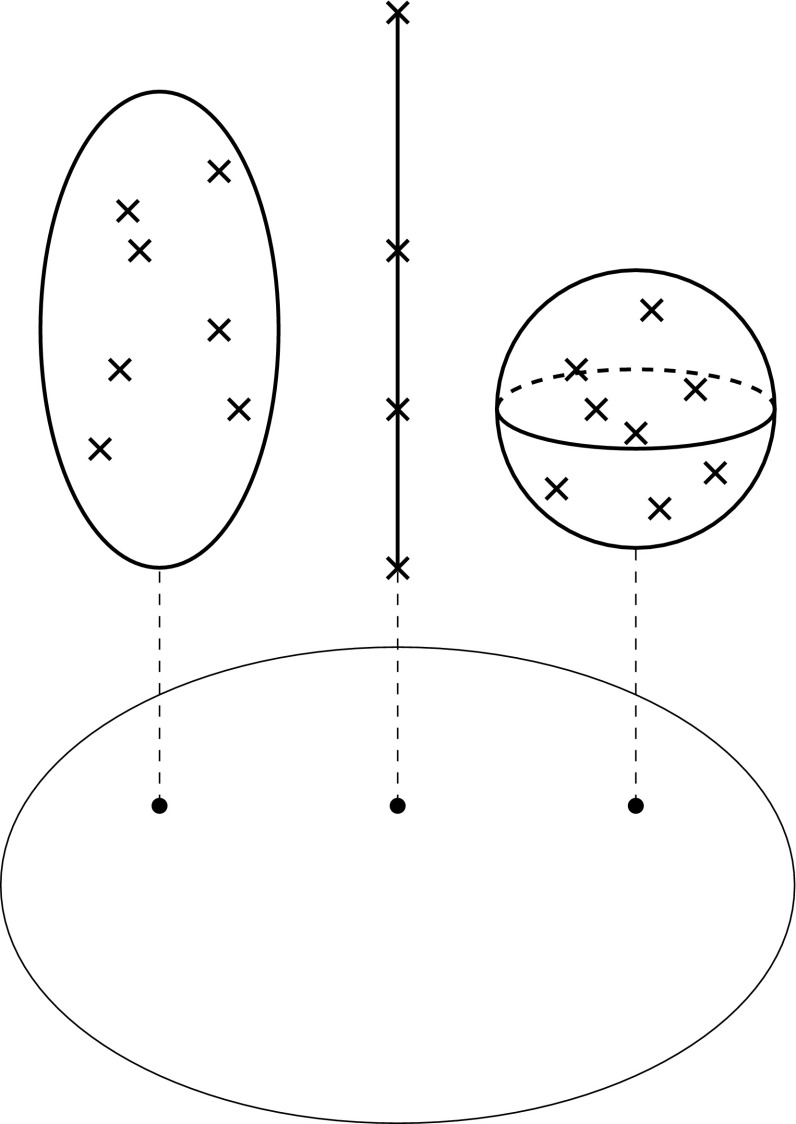
Fibers with different internal structures

**Fig. 3:**
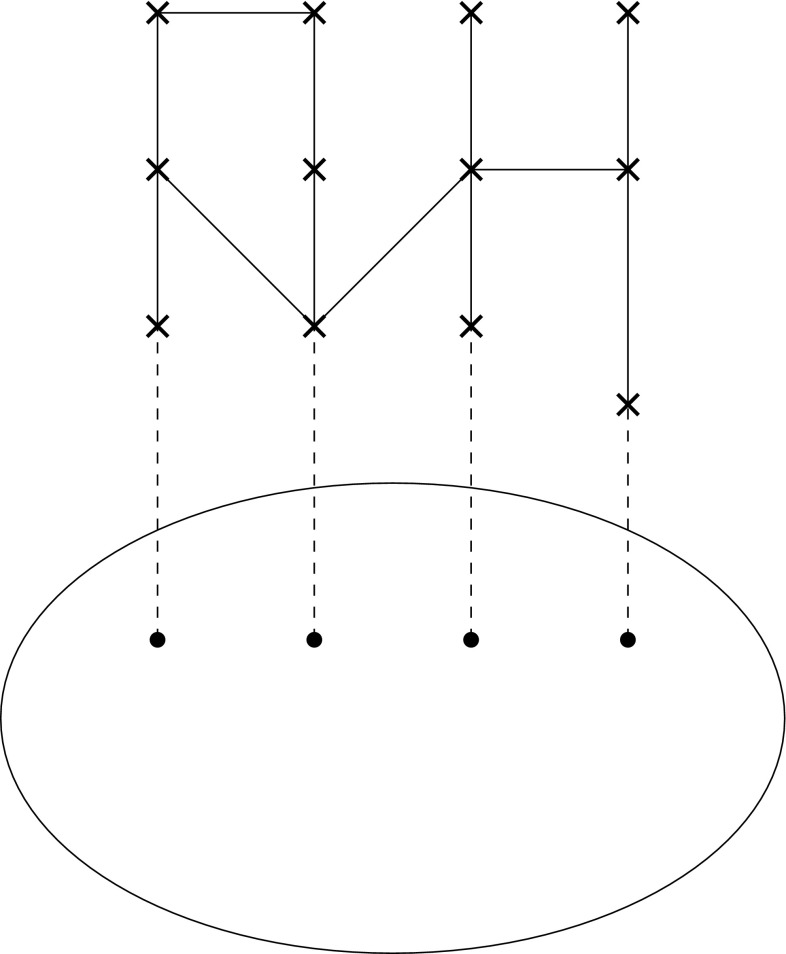
Partial section (*top*) and a global section

**Fig. 4:**
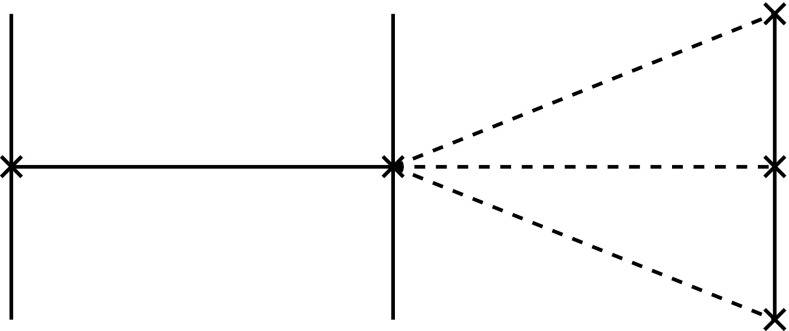
Partial section (*left*) with three possible extensions to a global section

**Fig. 5:**
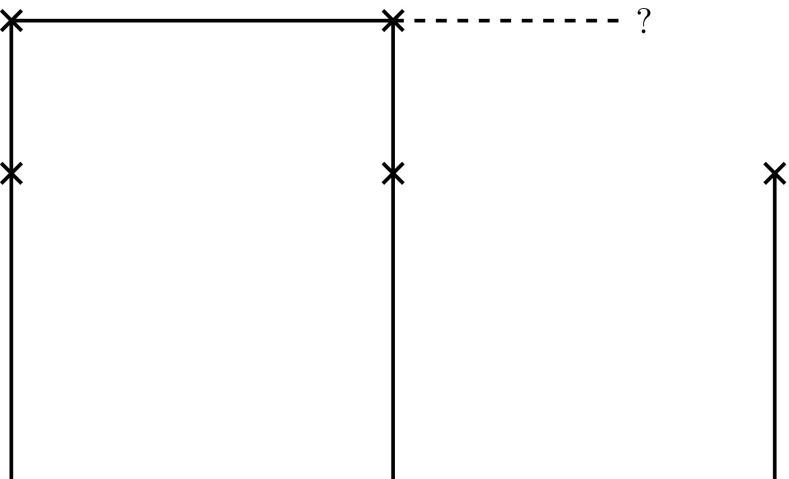
Partial section without an extension to a global section

We then formulate the following proposition for the representation of biological taxa in this geometric framework (Figs. 1, 2, 3, 4, 5).

### **Proposition 1:**

*A biological taxon corresponds to a partial section of the total space of all features under consideration. When that section is extended as far as possible, that is, when feature values (or possibly ranges) are assigned to all features that the members of that taxon possesses, then we have a species. Conversely, restricting the section to a smaller base corresponds to passing to a higher taxon, as this means that some feature values become undetermined. And thus, extending a section over a larger base means to pass to a more specific taxon. When there is more than one extension, then we have apomorphies, that is, feature values that can distinguish a taxon from a sister taxon. However, more specific taxa can also arise by an innovation as discussed below.*

*Since not all feature combinations can be represented by sections corresponding to biological taxa, the valid feature combinations correspond to structural, functional, developmental, or environmental constraints.*

Several remarks are needed for this proposition. “Under consideration” introduces a subjective moment, insofar as it is up to the biologists to select those features that they evaluate. Of course, as already discussed earlier, the selection of feature must be guided by biological principles as well as by schemes of data analysis. The term “ranges” refers to the fact that within each species, there is typically some variability in the feature values. Only those feature values that are simultaneously possible can be linked by a section. That is, the set of sections represents the possible feature combinations. Of course, it is not always easy to decide which combinations could exist, but by chance do not occur or have never been realized, and which ones are impossible because of structural, functional, developmental, or environmental constraints. We shall explore below how differences between those types of constraints manifest themselves in our geometric picture.

The advantage of the geometric approach over the algebraic one is that it allows for a simple description and classification of evolutionary novelties. This is what we shall now explore, as in Ehrig et al. (2017) where this mathematical framework is used to analyze business innovations.

In the geometric picture, we can distinguish the following types of innovations and explore their consequences.

1. Extending the value range of a feature *j*. This means that the fiber $F_j$ is enlarged by new values. The important consequence is that this may make extensions of partial sections possible, because the new feature value can, perhaps, be combined with the values of the other features that *i* possesses, whereas the previous values of *j* were not compatible with them.
2. Adding a new feature $j_{m+1}$. Again, a previous partial section $s_i$ might extend to a value $f_{j_{m+1}}(i)$ and thereby define $s_i(j_{m+1})$.
3. Amalgamation. This means the combination of two (or, perhaps, even more) partial sections into a larger one by identifying different structures into a single feature. For instance, in some models of bird evolution, the reptile ancestors of birds had evolved feathers for thermoregulation and extended forelimbs for climbing in trees. These two structures could then be combined into a wing, enabling them to first glide and then to actively fly (this is the arboreal hypothesis about the origin of bird flight, which is still controversial, but see, for instance, Burnham et al. 2011 for some recent support). Wagner (2014), p. 134 f., discusses fusion of ancestral characters as a source of novelty.

The last type, amalgamation, relates to, but is also conceptually different from, some ideas expressed in Seilacher’s construction morphology (Seilacher and Gishlick 2015) and to the concept of exaptation introduced by Gould and Vrba (1982), as a structure that had not evolved for its current function, but rather as a byproduct of some other functional structure, but which could then be employed and further developed for a new function. We refer to (Gould 2002) for an extensive discussion of this concept. What we want to emphasize here is the novel combination of preexisting structures to secure and support a new function. In an exaptation, a structure that was already present and available, but, so far functionless, acquires a function. Seilacher distinguishes structures originating from fabricational noise that were originally nonfunctional, but have subsequently acquired some functions and innovations as switches between primary and secondary functions. In contrast, in an amalgamation, two structures that had already their own functions are combined for a new function that neither of them could have fulfilled individually. In either case, new functions emerge from preexisting structures.

## Characters and phylogenetic trees

According to the prescriptions of Hennig’s cladism (1966), monophyletic groups are identified by synapomorphies, that is, shared derived characters. While this is conceptually as clear as it could possibly be, there exist some problems, as already mentioned in the introduction. Examples of reversion or convergence of characters have frequently been discussed in the literature. The Hox genes (Quiring et al. 1994; Gehring 1998) constitute another instance where phylogenetic relationships cannot be solely derived from phenotypic characters, because homologous Hox genes can control the development of morphological structures that themselves need not be homologous. Further such issues are discussed by Minelli (2003), p. 223ff, and he suggests a factorial view of homology and cites the work of Breidbach and Kutsch (1990) on the neuronal control of the dorsal longitudinal muscles in different body segments of both juvenile/larval and adult locusts and beetles. Breidbach and Kutsch identified a common set of 11 neurons with the same basic structure of dendritic fields that achieved that control in different segments (serial homology), between the different life stages of the same species and between different species. From this work, Olaf Breidbach (private communication) drew the consequence that one and the same basic neuronal control structure can be coupled with very different appendices in different insect species to carry out a wide range of different functions, and in his opinion, the flexibility achieved by coupling such a universal control structure with whatever actuator is needed for a specific task or in any given environment constitutes one of the reasons for the evolutionary success and adaptability of insects.

Gould (2002) argues that as a consequence of parallelism, that is, “independent origin channeled by common internal constraints of homologous genes or developmental pathways” (Gould 2002, p. 1074), “organismal branching” may occur “before gene branching”, and consequently, cladistic trees, as based on shared derived phenotypic characters, need not completely correspond to organismic trees (p. 1079). Conversely, serial homology in the sense of Owen might instantiate gene branching before organismal branching; such gene branching by itself cannot be used for the reconstruction of phylogenetic relationships between species, because not paralogs, the products of gene duplication, be they found in one and the same or in two different organisms, but only orthologs are special homologs that can be used for that purpose (ibd.).

**Fig. 6:**
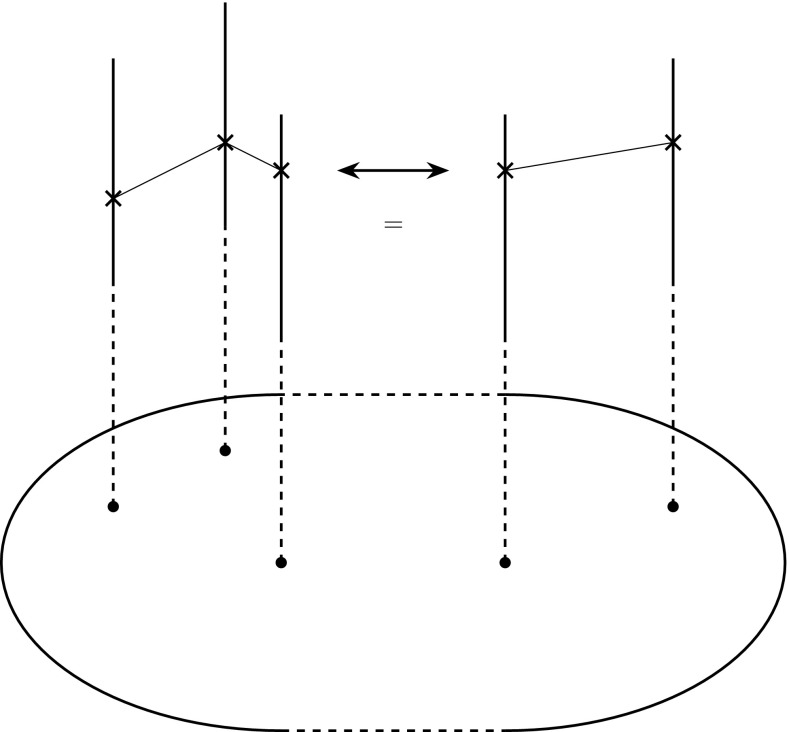
Amalgamation

The preceding considerations amplify these possibilities. When a structure is available to acquire a new function (exaptation) or can be combined with other such structures in a novel manner (amalgamation), then this can happen in parallel and independently within the phylogenetic group that possesses them. Thus, according to these possibilities, it is conceivable that not all terrestrial vertebrates descend from the first animal that had limbs to move on land, but that limbs developed independently in several descendants of an ancestor that already possessed the structural mechanisms for limb development, but which lay dormant until utilized through exaptation or amalgamation in those descendants. The corresponding characters may be difficult to detect in the fossil record.^7^ Such structures being available for being exapted and amalgamated might also underlie the punctuated equilibria that Eldredge and Gould (1972) had pointed out in the paleontological record (Fig. 6).

## Functional vs. phylogenetic correlations

We can now utilize the different schemes of representing correlations to translate the difference between functional and phylogenetic classifications into formal ones.

We shall discuss cladistic schemes, because they best highlight the contrast, but phenetic ones would likewise succumb to our treatment.

A cladistic character table can be represented in the form of a carrier-feature matrix as in (5), that is

21 $$\begin{aligned}&{\scriptstyle \,\,\,\,\,{\mathrm {character}\, 1}} \, \ldots \quad {\scriptstyle \mathrm {character}\,5}\nonumber \\ \begin{array}{c} {\scriptstyle \mathrm {species}\, 1} \\ \\ \vdots \\ \\ {\scriptstyle \mathrm {species}\, 5} \end{array}&\quad\left( \begin{array}{ccccc} 1 &{} 0 &{} 0 &{}0 &{}0 \\ 1 &{} 1 &{} 0 &{}0 &{}0 \\ 1 &{} 1 &{} 1 &{}0 &{}0 \\ 1 &{} 1 &{} 1 &{}1 &{}0 \\ 1 &{} 1 &{} 1 &{}1 &{}1 \end{array} \right) \end{aligned}.$$

Here, the last species possesses all the characters investigated. The fifth character is an apomorphy with respect to species 4, and the fourth is a synapomorphy of species 4 and 5 with respect to the other species, and so on. Thus, for instance, species 4 and 5 form a monophyletic group that has branched off from the rest. We point out that this cladistic matrix (21) has maximal rank ($=5$ in this example).

In contrast, a character matrix based on functional correlations would rather look like

22 $$\begin{aligned}&{\scriptstyle \,\,\,\,\,\,\,{\mathrm {character}\, 1}}  \ldots \quad {\scriptstyle \mathrm {character}\, 4}\nonumber \\ \begin{array}{c} {\scriptstyle \mathrm {taxon}\, 1}\\ \\ \vdots \\ {\scriptstyle \mathrm {taxon}\, 4} \end{array}&\quad\left( \begin{array}{cccc} \alpha _{1}&{} \alpha _{2}&{} \alpha _3 &{} \alpha _4\\ \beta _{1}&{} \beta _{2}&{} \beta _3 &{} \beta _4 \\ \gamma _{1}&{} \gamma _{2}&{} \gamma _3 &{} \gamma _4\\ \delta _{1}&{} \delta _{2}&{} \delta _3 &{} \delta _4 \end{array} \right) . \end{aligned}$$

Here, each taxon has its own characteristic set of characters which are correlated, as indicated by the common symbol utilized for the characters of each species. Therefore, here, the rank may be significantly lower ($=1$ in (23) below) than the maximally possible one ($=4$ in this example). This depends, however, on assignments that might look somewhat arbitrary. For instance, if taxon 1 is a carnivore, 2 a herbivore, 3 an insectivore, and so on, and if character 1 stands for the teeth, 2 for the feet, 3 for the digestive system, etc, we could label all the carnivore type character values as 1, the herbivore ones as 2, the insectivore ones as 3, to get the matrix

23 $$\begin{aligned}&{\scriptstyle \,\,\,\,\,\,\,\,\,\,\,{\mathrm {teeth}}}, {\scriptstyle \mathrm {feet}}, {\scriptstyle \mathrm {stomach}} \dots \nonumber \\ \begin{array}{c} {\scriptstyle \mathrm {carnivore}}\\ {\scriptstyle \mathrm {herbivore}}\\ {\scriptstyle \mathrm {insectivore}}\\ \dots \end{array}&\quad\left( \begin{array}{cccc} 1&{}1&{}1 &{}1\\ 2&{}2&{}2&{}2 \\ 3&{}3&{}3&{}3\\ &{} \dots &{} &{} \end{array} \right) . \end{aligned}$$

Of course, we have to realize that this scheme already presupposes the classification according to functional correlations. However, when we have large samples from many different taxa, we could cluster the features on the basis of their empirical correlations as the foundation of such a labelling. Thus, in the presence of large empirical data sets, one should first employ clustering algorithms and other machine learning tools to identify the character clusters and then try to neatly arrange them in a table like (22). In passing, we observe that this goes beyond the phenetic approach Michener and Sokal 1957 which originally wanted to treat all characters equally. Modern machine learning, in contrast, emphasizes the need for preprocessing, weighting, and clustering of data sets.

In particular, several functional structures may become combined, and then, instead of a tensor of rank 1 as in (23), we would get a higher rank. For instance, one group of features may derive from the functional needs of a carnivore, and another group from the requirements imposed by a cold climate. Thus, the algebra can disentangle different functional structures or clusters. In the following matrix, we distinguish between two types of feeding, carnivore (character value 1) and herbivore (2), and three climatic zones, arctic (1), temperate (2), and tropical (3), as represented in the following matrix:

24 $$\begin{aligned}&\qquad {\scriptstyle \mathrm {teeth}}, {\scriptstyle \mathrm {feet}}, {\scriptstyle \mathrm {fur}}, {\scriptstyle \mathrm {color}} \nonumber \\ \begin{array}{c} {\scriptstyle \mathrm {arctic\ carnivore}}\\ {\scriptstyle \mathrm {temperate\ carnivore}}\\ {\scriptstyle \mathrm {tropical\ carnivore}}\\ {\scriptstyle \mathrm {arctic\ herbivore}}\\ {\scriptstyle \mathrm {temperate\ herbivore}}\\ {\scriptstyle \mathrm {tropical\ herbivore}} \end{array}&\qquad \left( \begin{array}{cccc} 1&{}1&{}1 &{}1\\ 1&{}1&{}2&{}2 \\ 1&{}1&{}3&{}3\\ 2&{}2&{}1 &{}1\\ 2&{}2&{}2&{}2 \\ 2&{}2&{}3&{}3 \end{array} \right) \nonumber \\ \\&= (1,1,1,2,2,2)\otimes (1,1,0,0)+ (1,2,3,1,2,3)\otimes (0,0,1,1) \end{aligned}$$

which has rank 2, as the overlay of two character clusters. Again, we should emphasize that the labelling of the characters by numbers is arbitrary, and arranged here for convenience. The value 2 for teeth has nothing to do with the value 2 for fur, for instance, as these are mere labels. The only relevant aspect is that there are two types of systematic correlations. All carnivores have the same labels for feet and teeth, and all arctic animals have the same labels for fur and color, for instance. Of course, the algebra of this example is rather artificial, but it is not difficult to see the pattern.

More easily and transparently, however, much of the preceding can be represented in the geometric framework described above. Sections of a presheaf corresponding to a cladistic branching then also branch off from each other, whereas those corresponding to functional clusters tend to be disjoint from each other.

When we utilize the geometric representation, we can also invoke the geometric gestalt concept developed in (Breidbach and Jost 2006). The gestalt concept was originally conceived by von Ehrenfels (1890) in cognitive psychology as the basis of the invariant recognition of patterns. He formulated two basic criteria for a gestalt, superadditivity and transposability. Superadditivity means that a gestalt is more than the sum of its parts, and this leads to its priority over those individual parts in perception. An example for the second criterion, transposability, is a melody that preserves its identity independently of the key or tune. Both aspects are also relevant clearly in morphology where one wants to recognize distinctive patterns that are invariant under certain types of transformations.

This will also provide a mathematical foundation for the theory of the botanist Troll (1928) who saw the purpose of morphology in the transformation of patterns (gestalten, but we employ that term in a somewhat different sense), as well as a firmer foundation for the theory of Thompson (1917).

For that purpose, we assume that the fibers $F_j$ admit actions of a symmetry group *G*, that is, for each $g\in G$, we have an operation

25 $$\begin{aligned} g:F_j \rightarrow F_j \end{aligned}$$

with

26 $$\begin{aligned} (g\circ h)(x)=g(h(x))\; \text { for\, all }\;g,h\in G\; \text { and\; each }\; x\in F_j, \end{aligned}$$

where on the left-hand side, we have the multiplication $g\circ h$ in the symmetry group *G*, and on the right hand side, we have the composition of self-maps of $F_j$. For instance, *G* could be simply the scaling group. When the overall size of an animal is decreased, as in certain dwarf species, like *Choeropsis* (or *Hexaprotodon*) *liberiensis*, then all body parts need to be shrunk correspondingly, and this is precisely what our formalism attempts to capture.

The operation of the groups *G* could be the same for each fiber $F_j$ or could depend on that fiber.^8^ It is also possible that on some of the fibers, the group operates trivially, that is, $g(x)=x$ for all *x*. We can then also apply the group operation to a section *s*, by putting

27 $$\begin{aligned} g(s(j))(x)=g(s(j)) \end{aligned}$$

where on the right hand side, *g* operates on the element $s(j) \in F_j$.

The group *G* then yields a gestalt in the sense of (Breidbach and Jost 2006):

### **Definition 2**

A gestalt is defined as the invariants of a collection of patterns that can mutually be transformed into each other through the elements of a transformation group *G*.

The group *G* here could consist of scalings or other geometric deformations, as in (Thompson 1917), or of reflection or rotation symmetries.

**Fig. 7:**
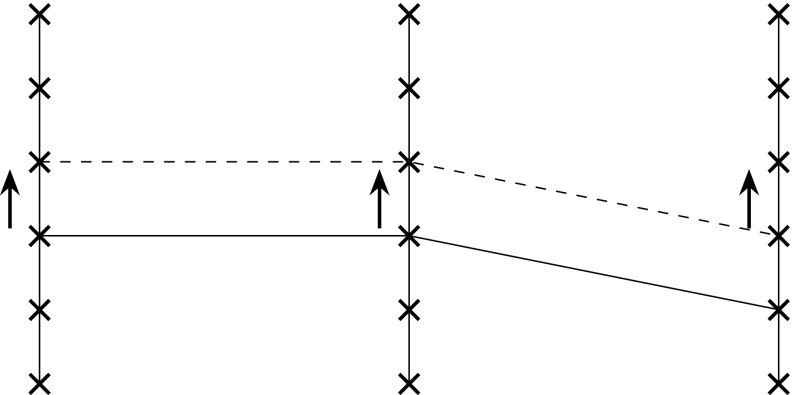
Translation of a fiber as an example of a symmetry

The group transformations could express relations between different taxa, transforming one into another taxon, or they constitute internal symmetries, like the bilateral symmetry of bilaterians. Group theory also provides tools for the description of the unfolding of a structure in embryonic development, but for that purpose, it should be combined with the theory of dynamical systems (see Jost 2014), following Thom (1975). In biological examples, of course, the group laws need not be fully satisfied. For instance, serial homologies like between the vertebrae are encoded by transpositions, but only on a finite chain, the vertebral one, instead of on an infinite chain or a closed loop as required for a translation group. In addition, with the help of the concept of a transformation group, we can also explore the non-realized parts of the morphospace in the sense of (Raup and Gould 1974) (Fig. 7).

## Footnotes

1 This contribution owes much to the discussions that I have had with my friend Olaf Breidbach over several decades, on this and many other topics, to his deeply penetrating questions, his vast knowledge of the history of biology, his deep insight into the structure of biological theory, and most of all, his profoundly original way of thinking.

2 Goethe had used the term “Morphologie” in his writings since 1796, but published it only in 1817. In 1800, K.F. Burdach had introduced the term apparently independently of Goethe, see Burdach (1800); Nyhart (1995) and the entry “Morphologie” in Toepfer (2011).

3 There is some ironic twist here, as the turtle carapace is actually formed from or spanned by bones that are the homologues of the ribs of other vertebrates, see Klein and Kuraku (2011) and the discussion in Wagner (2014), p. 179ff., as well as the paleontological references given in Benton (2015), p. 243

4 Oken had visited Geoffroy 1821 in Paris and collaborated with him on the analysis of developmental processes. Later, he had some influence on Richard Owen, see Breidbach et al. (2002).

5 It might be conceivable that also the variability of some particular character is characteristic for a particular species, but we shall not explore that possibility here. In addition, in any case, variability is not a property of individuals, but of populations.

6 The historical facts are somewhat subtle. I do not wish to enter the debate here, but only mention that Goethe did discover the intermaxillary bone in humans, but the general comparative analysis is due to Geoffroy St. Hilaire (1818) and Meckel (1820). Some references are Russell (1916); Opitz (2004); Takechi and Kuratani (2016); Amundson (2005); Toepfer (2011).

7 For an example of the current state of fossil evidence for the water–land transition, see, for instance, Ahlberg and Clack (2006).

8 Alternatively, we might assume that on each fiber, a different group operates. Mathematically, this amounts to the same.
